# Neuronal synaptic architecture revealed by cryo-correlative light and electron microscopy

**DOI:** 10.52601/bpr.2024.240035

**Published:** 2025-06-30

**Authors:** Pei Wang, Buyun Tian, Xiaojun Xu, Huiqin Luan, Yan zhang, Wenhao Sun, Liqiao Hu, Yuanyuan Li, Yuchen Yao, Weixing Li, Shuli Zhang, Xia Li, Wei Feng, Wei Ji, Yanhong Xue

**Affiliations:** 1 Key Laboratory of Biomacromolecules (CAS), National Laboratory of Biomacromolecules, CAS Center for Excellence in Biomacromolecules, Institute of Biophysics, Chinese Academy of Sciences, Beijing 100101, China; 2 Guangzhou National Laboratory, Guangzhou 510005, China; 3 University of Chinese Academy of Sciences, Beijing 100049, China; 4 National Research Center for Rehabilitation Technical Aids, Beijing 100176, China; 5 State Key Laboratory of Brain and Cognitive Sciences, Institute of Biophysics, Chinese Academy of Sciences, Beijing 100101, China; 6 Institute of Special Environmental Medicine, Co-innovation Center of Neuroregeneration, Nantong University, Nantong 226001, Jiangsu, China

**Keywords:** Cryo-CLEM, Cryo-ET, Presynaptic, Synapse structure, Synaptic cleft

## Abstract

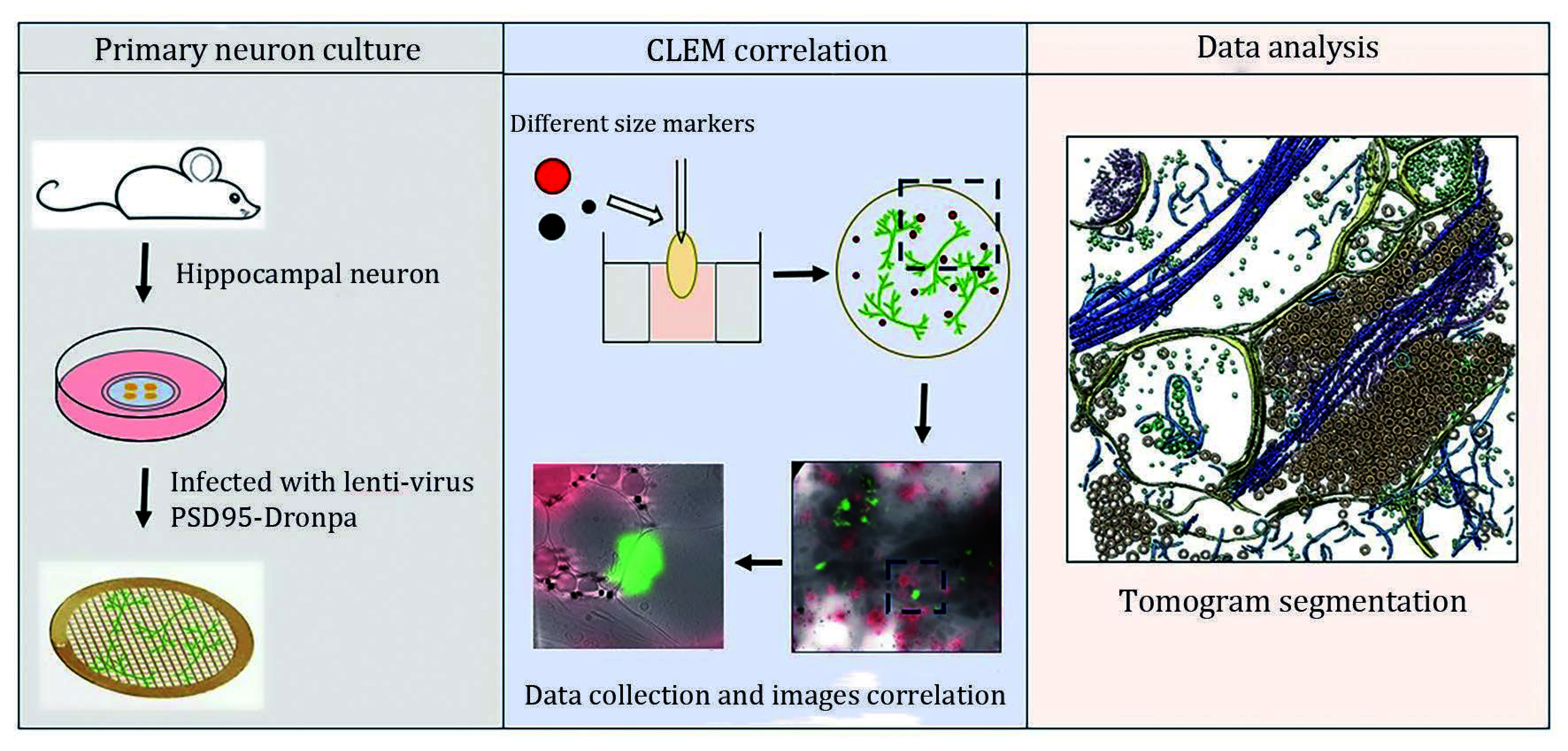

Cryo-correlative light and electron microscopy (cryo-CLEM) is a powerful technique that combines fluorescence imaging for specific localization with electron microscopy for detailed structural analysis, enabling high-resolution exploration of synaptic structures in neurons. In this study, we employed a cryo-CLEM approach using three independent alignment markers to precisely correlate electron microscopy (EM) images with light microscopy (LM) images of neuronal synapses under cryogenic conditions. This methodology revealed a distinctive pattern of electron densities in the synaptic clefts. Additionally, we were able to capture high-resolution images of presynaptic vesicles in various states, underscoring the potential of cryo-CLEM in advancing synaptic research.

## INTRODUCTION

Synapses, which serve as specialized connections between neurons, are essential for communication and the formation of neural circuits that underpin all brain activities (Alonso-Nanclares *et al.* 2008; Mayford *et al.* 2012; Sudhof and Malenka 2008). Chemical synapses consist of the presynaptic terminal, the postsynaptic membrane, and the synaptic cleft between them (Burns and Augustine 1995; Gray 1959). Within the presynaptic terminal, synaptic vesicles, typically 30–50 nm in diameter, contain neurotransmitters and are capable of fusing with the presynaptic membrane (Li *et al.* 2019; Zhang *et al.* 1998). The postsynaptic membrane is rich in receptor proteins and ion channels, which mediate either excitatory or inhibitory responses (Cohen-Cory 2002). The synaptic cleft, the extracellular gap between the presynaptic and postsynaptic membranes, is approximately 20–30 nm in width (Tao *et al.* 2018). Studying the ultrastructure of synapses is critical for understanding the mechanisms of synaptic transmission. Synaptic dysfunction is associated with various neurodevelopmental and neuropsychiatric disorders, such as Alzheimer’s disease (Blanco-Suárez *et al.* 2017; Peng *et al.* 2022; Scheff *et al.* 2015), schizophrenia (Blanco-Suárez *et al.* 2017; Yin *et al.* 2012), depression (Duman and Aghajanian 2012; Duman *et al.* 2016) and autism (Spooren *et al.* 2012). Thus, detailed characterization of synaptic structures is essential for advancing our understanding of their functional mechanisms.

The resolution of conventional optical microscopes is insufficient to reveal the ultrastructure of synapses, while electron microscopes with angstrom-level resolution can achieve this goal, enabling the observation of detailed synaptic ultrastructure and the determination of its morphology (Imig *et al.* 2014; Siksou *et al.* 2007). Under conventional electron microscopy (EM), synapses exhibit distinctive characteristics in both the presynaptic and postsynaptic regions, with electron-dense materials near the plasma membrane serving as crucial components (Gray 1963; Imig *et al.* 2014; Phillips *et al.* 2001; Sudhof 2012). Conventional EM observations have contributed to our current understanding of the ultrastructure of synapses, such as the postsynaptic density (PSD), and the synaptic vesicles at the active zone. However, the conventional EM sample preparation procedures typically entail dehydration, chemical fixation, heavy metal staining, and plastic embedding, which introduce sample artifacts and lead to inconsistent results from different sets of experiments (Imig *et al.* 2014; Korogod *et al.* 2015). To address these challenges, cryo-electron tomography (Cryo-ET) has become the preferred technique for studying cellular structures of synapses at nanometer resolution. Since no heavy metal stains or contrast agents are utilized, the resulting contrast directly reflects biological density, allowing for 3D visualization of fully hydrated, unstained cells. This approach preserves structural details far better than conventional EM techniques (Li *et al.* 2023; Mahamid *et al.* 2016; Tao *et al.* 2018; Wang *et al.* 2021).

Cryo-ET presents additional challenges in sample preparation. As specimens are embedded in vitreous ice, they are more difficult to slice mechanically compared to samples embedded in plastic. Despite this, vitreous sectioning has been employed in synaptic studies of mammalian organ slices (Fernandez-Busnadiego *et al.* 2010; Zuber *et al.* 2005), with drawbacks such as low throughput and artifacts like substantial compression along the cutting direction (Fernandez-Busnadiego *et al.* 2010). Isolated synaptosomes are also frequently analyzed by cryo-ET, as they can form an appropriate ice thickness on EM grids without sectioning, which facilitates high-throughput data acquisition. However, synaptosomes may not consistently release neurotransmitters, and alterations to the presynaptic cytoskeleton during sample preparation can cause deviations from the physiological state (Fernandez-Busnadiego *et al.* 2010; Imig *et al.* 2014). Therefore, primary neurons grown directly on EM grids are more suitable for synapse study in cryo-ET imaging (Asano *et al.* 2015; Lucic *et al.* 2007; Tao *et al.* 2018; Wang *et al*. 2021). This method allows for imaging of neurites without sectioning or thinning, preserving the *in-situ* structure of presynaptic terminals, postsynaptic membranes, and the synaptic cleft. Unlike chemically fixed samples, the postsynaptic density area of vitrified neurons is not as obvious as in conventional EM (Kaeser *et al.* 2011), Thus, the new challenge of cryo-ET studies lies in identifying and localizing synapses in primary neurons.

Fortunately, cryo-correlative light and electron microscopy (cryo-CLEM) integrates the specificity of fluorescent labeling and the high resolution of electron microscopy, allowing us to utilize specific fluorescent markers to target and localize proteins under cryogenic conditions (Chang *et al.* 2014; Liu *et al.* 2015; Sartori-Rupp *et al.* 2019; Tao *et al.* 2018). Another key step in cryo-CLEM is the correlation of light microscope (LM) images with EM images. In this study, we leveraged a cryogenic optical microscopy imaging platform developed in our lab (Liu *et al.* 2015; Xu *et al.* 2018) and used the classic synaptic protein PSD95 as the marker to identify the synapses in cultured primary hippocampal neurons. To precisely align the LM and EM images across multiple magnifications, we employed three independent alignment markers. These markers, each with different sizes, enabled accurate correlation and revealed the ultrastructure of synapses in their native states.

## RESULTS

### Preparation of frozen-hydrated primary hippocampal neurons on EM grids

**Figure 1:**
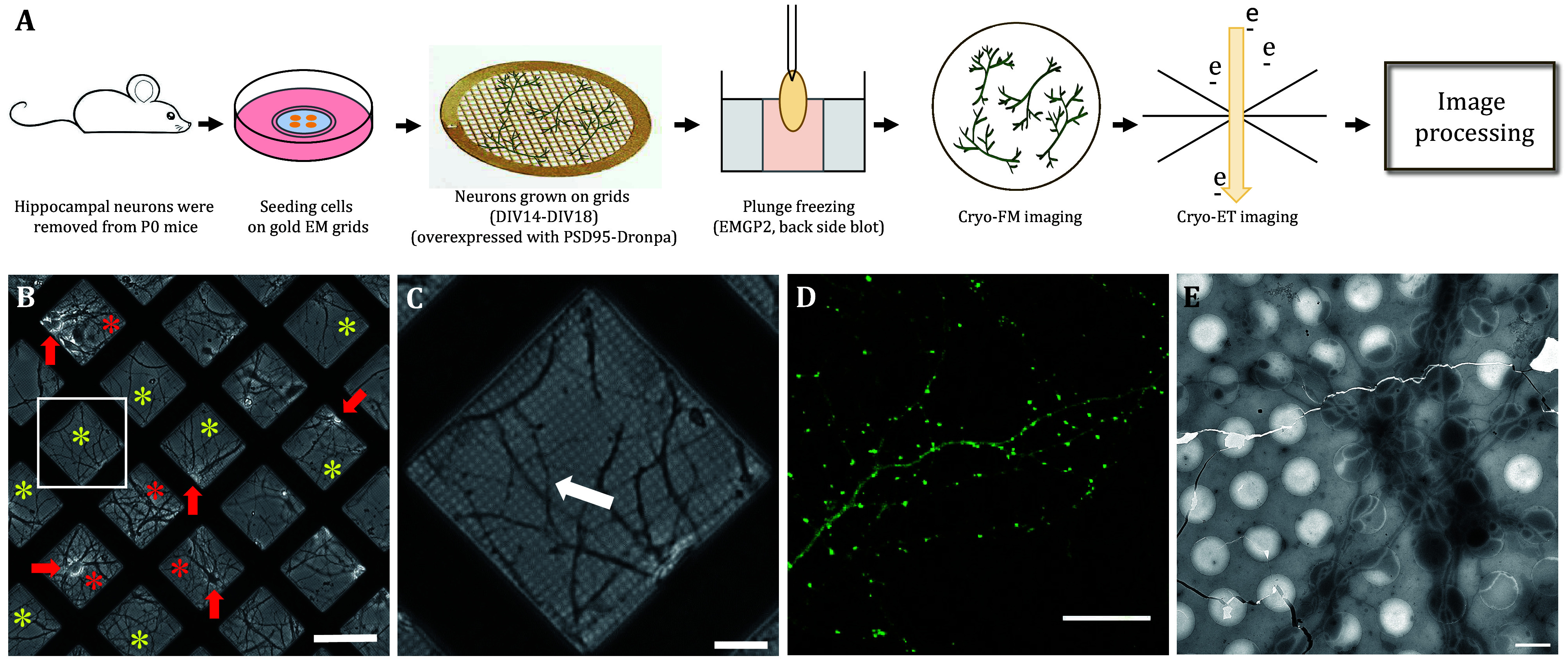
Visualization and vitrification of primary hippocampal neurons overexpressed PSD95-Dronpa grown on EM grids. **A** Workflow of cryo-ET/cryo-CLEM of primary neurons grown on EM grids. **B** Light microscopy (LM) image of cultured neurons at DIV16 on PDL-coated EM grids. Yellow asterisks represent the suitable square for cryo-ET data collection, red asterisks indicate that the neurites are too dense for imaging, and red arrows indicate the neurons’ soma area. **C** Zoom-in view of white boxed regions in Panel B. White arrows represent neurites. **D** Fluorescence image of PSD95 labelled Dronpa of neuron cells. PSD95 is a typical excitatory postsynaptic marker. **E** Low magnification TEM image shows the vitrified neurons on EM grids. Scale bars: 10 μm (**B**), 2 μm (**C**), 20 μm (**D**), 2 μm (**E**)

To visualize the ultrastructure of synapses from primary hippocampal neurons, we directly seeded dissociated primary neurons onto EM gold grids for low-density culture until the neurons matured in 14–16 days. After ten days of culture *in vitro*, primary hippocampal neurons contain dendrites and axons, and the presynaptic varicosities and excitatory connections are established. Inhibitory synapses, constituting approximately 25% of all synapses in hippocampal culture, typically form at a later stage (Grabrucker *et al.* 2009). To evaluate whether the neurons are healthy and suitable for cryo-ET imaging, we examined them under a light microscope. The cell body appears smooth and bright, and the neurites extend well without being broken, covering most of the grid squares and forming a stable neuronal network (Figs. 1B and 1C). Subsequently, cells were plunged freezing into liquid ethane to obtain the vitrified sample (Fig. 1E).

Frozen-hydrated samples were not chemically fixed or stained, resulting in low image contrast in cryo-EM. This limitation compromises the identification of synapses during cryo-ET data collection. Therefore, we utilized lentivirus to overexpress PSD95-Dronpa, specifically labeling excitatory synapses (Fig. 1D displays fluorescent puncta of PSD95-Dronpa at DIV16 in hippocampal neurons). To facilitate the correlation of cryo-FM and cryo-ET images, 200 nm fluorescent microspheres and gold particles of different sizes (10 nm and 50 nm) were deposited to sample when plunging freezing. The soma and dendritic regions are too thick to allow electrons to penetrate through, resulting in imaging failure or extremely poor image contrast. In contrast, axons are thinner and thus more amenable to cryo-ET imaging.

### Correlation of cryo-CLEM images with three alignment markers

**Figure 2:**
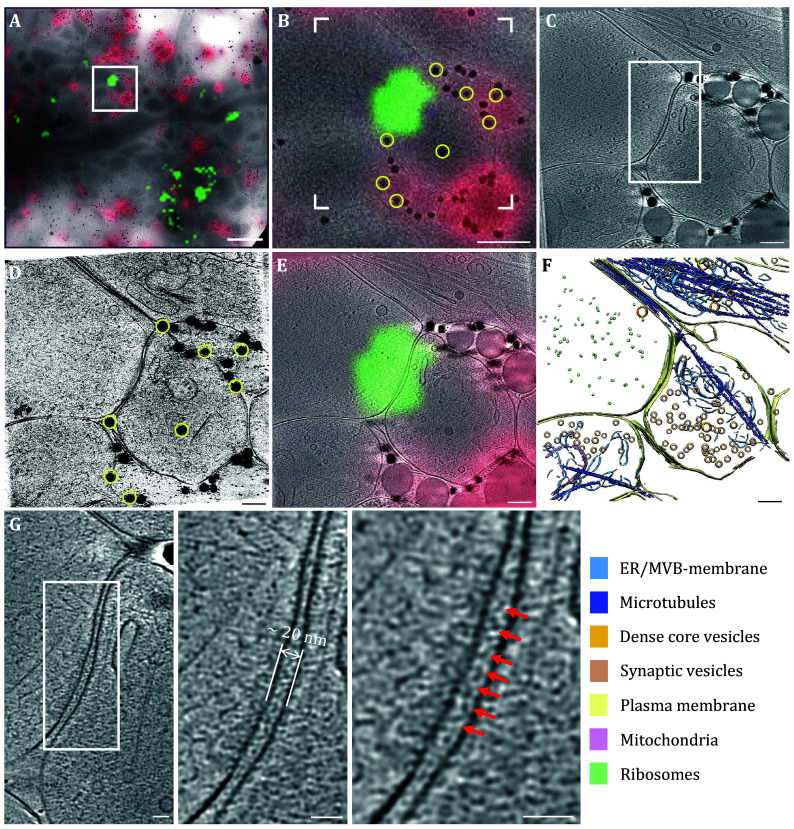
Fine correlation of cryo-FM and tomographic slice at high-magnification shows that synaptic cleft has a dense band in PSD95-Dronpa labelled neurons. **A** Overlay cryo-FM and cryo-EM images. Green represents the PSD95-Dronpa signal, red indicates the 200 nm dark red fluorescent microsphere signals. **B** Zoom-in view of white boxed area in Panel A. White dashed square indicates the tilt-series collected area according to the PSD95-Dronpa signal (green). **C** A tomographic slice of the white dashed square in Panel B, showing a synapse acquired by cryo-ET. **D** 2D projection of tomogram in Panel C along the z direction. Yellow circles represent the 50 nm gold particle used for fine correlation of cryo-FM and tomographic slice at high-magnification. **E** Overlaid cryo-FM and cryo-EM image shows a synapse colocalized with PSD95-Dronpa puncta. **F** Segmentation of the tomogram shown in Panels C and E. **G** Enlarged views of synaptic cleft shown in Panel C, displayed a dense band of electrons in the cleft about 20 nm wide. Scale bars: 2 μm (**A**), 500 nm (**B**), 200 nm (**C**, **D**, **E**, **F**),50 nm (**G**)

Most cryo-CLEM procedures typically involve a three-step workflow between cryo-fluorescence microscopy (cryo-FM) and cryo-electron microscopy (cryo-EM) facilities. The first step entails performing fluorescence imaging in cryogenic conditions. The second step is acquiring EM images of the position of interest (POI) based on the fluorescence data. The last step is correlating the cryo-FM/EM images. Various techniques were used to facilitate the correlation of images at different magnifications. For example, the EM grid with coordinates (numbers or letters) greatly benefits the initial correlation at a low magnification. We used three markers of different sizes to correlate the cryo-FM and cryo-EM images at multiple scales. Dark red fluorescent microspheres with a diameter of 200 nm, capable of fluorescing in cryo-FM and appearing electron-dense in cryo-EM, were applied to correlate the FM image and EM montage map at a middle magnification (3600×) (supplementary Figs. S1C–S1G). 50 nm gold particles were utilized to merge the fluorescent signal with high-magnification tomographic slices (Figs. 2A–2E, and supplementary Movie S1). A 10-nm gold tracer was applied to do the tilt-series alignment.

Various steps of cryo-FM and cryo-EM/ET images were acquired as described in methods (Fig. 2, and supplementary Fig. S1). With the assistance of fiducial markers of different sizes, we successfully obtained overlayed images of cryo-FM and cryo-ET (Fig. 2E). The entire workflow of image processing is presented in supplementary Fig. S2. Ultimately, we collected 18 excitatory synapses, co-localized with PSD95-Dronpa, as identified by cryo-CELM.

### Ultrastructural characterization and organization of excitatory presynapses

**Figure 3:**
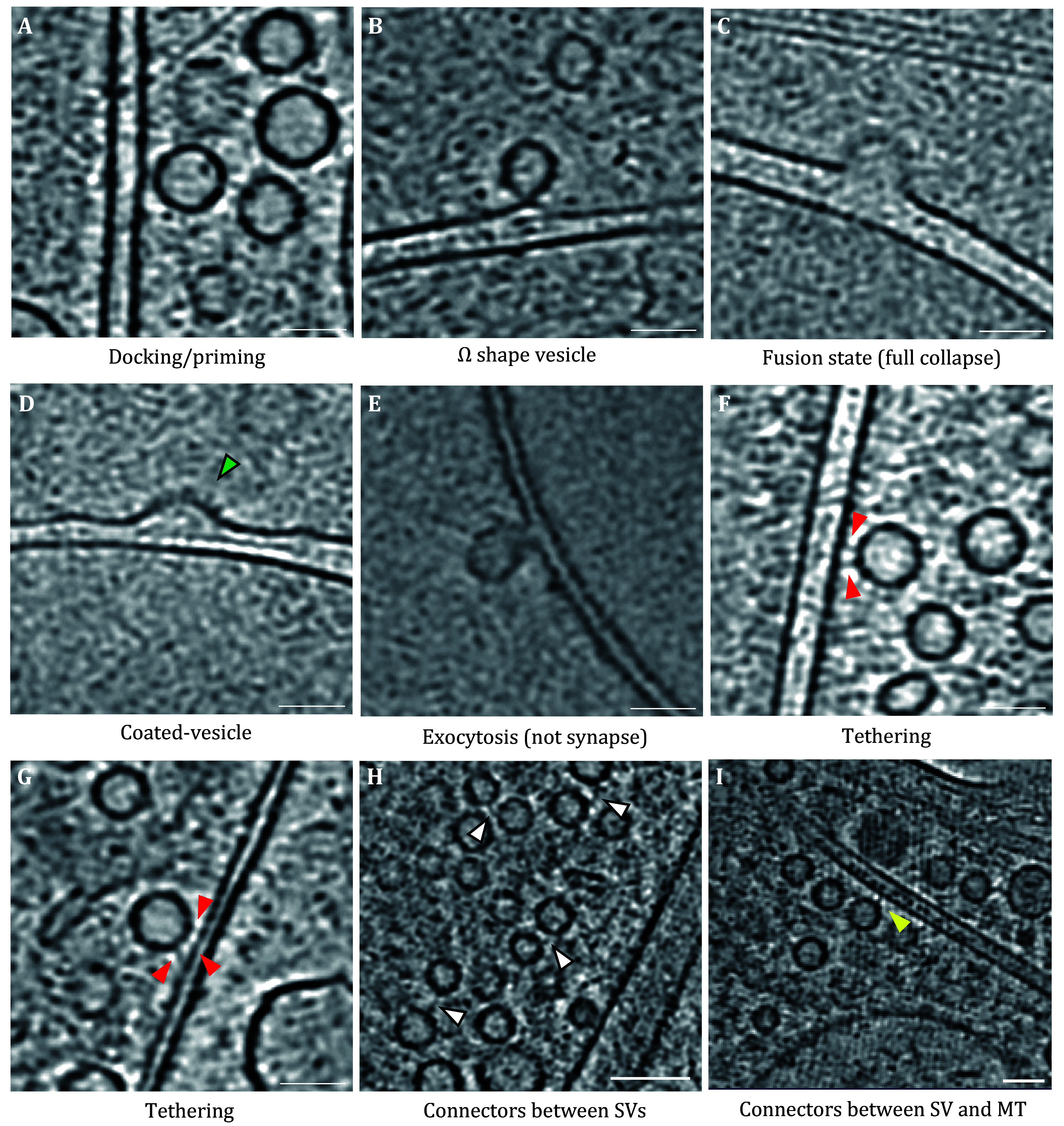
Synaptic vesicles in different states. **A** Vesicle is very close to the presynaptic membrane, in a docking or priming state. **B** Vesicle is fusing with the presynaptic membrane, in “Ω” shape. **C** Full collapse of the vesicle into the plasma membrane. **D** Coated-vesicle with a burry surface. The green arrowhead may refer to the clathrin. **E** Vesicle presents an "Ω" shape, and the exocytosis occurs in the none active zone. **F**,**G** Vesicles are tethered to the presynaptic membrane. The red arrowheads indicate the tether proteins. **H** Connectors linking two vesicles. White arrowheads indicate the connectors. **I** Connectors between synaptic vesicles and microtubules. The yellow arrowhead indicates the connector. Scale bars: 50 nm

We observed synaptic vesicles in various states, including vesicles docked at the presynaptic membrane or in priming state (Fig. 3A), vesicles fusing with presynaptic membrane in the shape of “Ω” (Fig. 3B), vesicle fully fused with the presynaptic membrane (Fig. 3C), and coated vesicle (Fig. 3D), maybe clathrin-mediated vesicle in the recycling state after fusion with the presynaptic membrane. There are also vesicles released in the non-synaptic active zone (Fig. 3E). These vesicles in various states also reflect that the primary hippocampal neurons we cultured have normal and healthy synaptic functions.

Furthermore, we also observed vesicles tethered to the presynaptic membrane through tether proteins (Figs. 3F and 3G). Previous studies based on chemical fixation and heavy metal staining showed that the presynaptic active zone (AZ) has distinct characteristics of electron-dense contents, where synaptic vesicles (SVs) accumulate and are released. However, our cryo-ET data suggested that these electron densities may be dehydration-collapsed tether proteins. In fact, we found that in cryo-ET data from plunging-freezing hippocampal neuronal synapse, vesicles directly in contact with presynaptic AZ are extremely rare and appear only during vesicle fusion. Most SVs near the AZ were connected to the membrane through the filament-like structure. Vesicles in the tethering state are more likely to undergo rapid release. Since most of these filament-like tether proteins are smaller than 10 nm, these structures may be covered by heavy metal staining or not observed, due to low resolution in previous conventional electron microscopy studies of synapses. Therefore, it is necessary to use high-resolution cryo-ET to study synapses in their near-physiological states.

Synaptic vesicles are highly organized in the presynaptic environment. We found that there are connectors between SVs. Vesicles may be connected to one or more vesicles through a single connector or multiple connectors, forming a dynamic SVs network (Fig. 3H). Some studies have previously speculated that the connectors between vesicles are composed of synapsin (Hirokawa *et al.* 1989), but synapsin-knockout samples showed the presence of connector structures (Siksou *et al.* 2007). Therefore, the molecular composition of connectors still needs to be explored. Additionally, we observed connections between the vesicle and microtubule (Fig. 3I), potentially related to the transport of synaptic vesicles within neurons. It is unclear whether the connections between SVs and between vesicle and microtubule have the same molecular compositions.

### Electron-dense in the synaptic cleft located on the postsynaptic membrane

In the 18 synapses identified by our cryo-CLEM, there are no obvious electron-dense in the presynaptic “active zone” area and postsynaptic PSD area (Fig. 2C). However, the synaptic cleft exhibits distinctive features. The edges of the synaptic cleft are smooth, and the cleft has a relatively uniform width of approximately 20 nm. Notably, there is significant electron density in the cleft with some patterns (Fig. 2G and supplementary Fig. S3).

Conventional electron microscopy studies of synapses often highlight a prominent electron-dense postsynaptic density near the postsynaptic membrane. However, under cryogenic conditions, we did not find the existence of electron-dense materials in the PSD region. Instead, we observed an increase in electron density within the synaptic cleft. We can vaguely see that these electron-dense substances seem to be closer and connected to the postsynaptic membrane.

**Figure 4:**
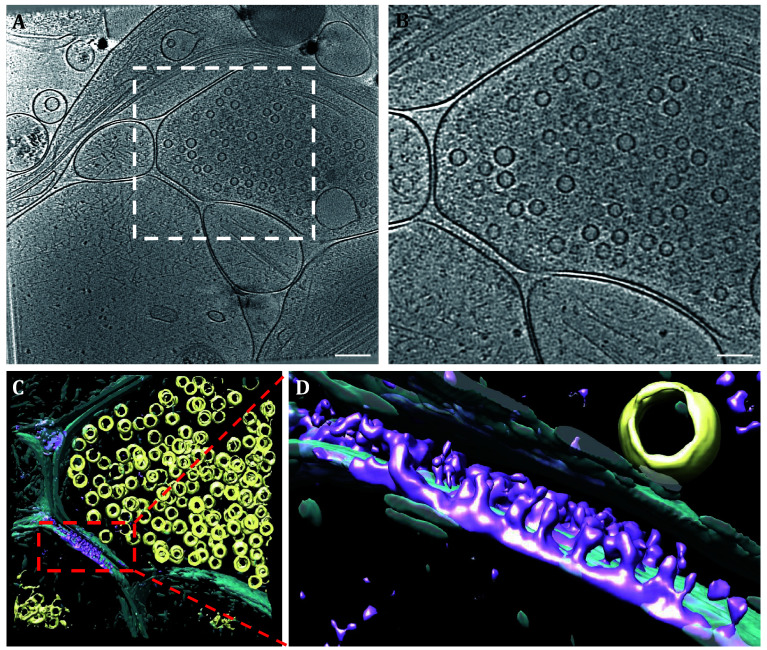
The electron-dense represented proteins located in the postsynaptic membrane. **A** A tomographic slice of a typical synapse. **B** Enlarged views of the synaptic cleft area (dashed box) shown in Panel A. **C** Sub-volume segmentation of tomogram in Panel A showing the electron-dense in synaptic cleft located in the postsynaptic membrane. **D** Enlarged view of red dashed box in Panel C. Yellow represents the SVs, cyan represents the synaptic membrane, and purple represents the interested proteins on the postsynaptic membrane. Scale bars: 200 nm (**A**), 100 nm (**B**)

To enhance the data quality, we performed noise reduction and contrast transfer function (CTF) correction to the three-dimensional reconstructed data. Algorithms were employed to restore missing-wedge angle information. The sub-volume of the synaptic cleft area was extracted and segmented, revealing that the proteins represented by the electron-dense materials in the synaptic cleft were exclusively situated on the postsynaptic membrane (Figs. 4C and 4D). The electron-dense proteins in the synaptic cleft were inserted vertically into the postsynaptic membrane, with no direct connection to the presynaptic membrane. Since there are a variety of receptor proteins for neurotransmitters and various cell adhesion molecules distributed on the postsynaptic membrane to help neurons communicate with each other, it is not surprising that these electron-dense substances in the synaptic cleft are actually postsynaptic membrane proteins.

## DISCUSSION

In the past, it was widespread and common to use conventional electron microscopy to study the distribution of synaptic vesicles in the synaptic terminal and the neurotransmitter release mechanism. However, there are often conflicting findings from different studies on the same issue (Imig *et al.* 2014), possibly due to the dehydration and deformation of the biological sample caused by chemical fixation. Therefore, cryo-ET imaging, which allows observation of frozen-hydrated biological samples, is the optimal way to study synapses in a near-physiological state. Since the PSD region is not visible under frozen conditions, we used cryo-CLEM to specifically identify excitatory synapses based on the fluorescent signal of PSD95-dronpa. To account for the absence of inhibitory synapses in this method, we also randomly collected unlabeled neuronal synapses and observed that the dense electron bands were present not only in the PSD95-Dronpa-labeled synaptic clefts but also in the unlabeled ones (supplementary Fig. S4 and Movie S2). This suggests that in frozen-hydrated samples, electron-dense regions in the synaptic cleft exist in both excitatory and inhibitory synapses.

Based on these observations, we propose several criteria for identifying synapses under cryo-conditions: (1) The presence of at least two spatially opposed membranous structures, with one containing a large number of synaptic vesicles (SVs) and the other containing few or no vesicles; (2) Some SVs are attached to the presynaptic membrane (in tethering/docking/priming or even fusing state); (3) The synaptic cleft exhibits a smooth edge, with a uniform width of about 20 nm, and contains electron densities within cleft. We believe these criteria for synaptic identification under cryogenic conditions, along with the cryo-CLEM workflow, will significantly enhance the application of cryo-ET in synaptic research.

We employed primary cultured hippocampal neurons, characterized by synaptic transmission and plasticity, as a model system to study synapses. While we acknowledge potential differences in synaptic maturity compared to the adult brain, the ultrastructural features observed in these neurons closely resemble those found in human neural networks. Our approach enables precise synaptic localization and provides high-resolution ultrastructural images, capturing various states of synaptic vesicles within the presynaptic terminal and numerous molecular receptors located on the postsynaptic membrane within the synaptic cleft. Studying the distribution of SVs and the readily releasable pool (RRP) or release quantification under near-physiological conditions allows for drawing the most reliable conclusions, as it mitigates artifacts associated with sample preparation methods. Furthermore, significant advances in resolving the *in-situ* structure of synapses with cryo-ET (Liu *et al.* 2020; Watanabe *et al.* 2020) suggest that high-resolution, in-situ analysis of synapse-associated proteins holds great promise for future studies.

## METHODS

### EM grids preparation

Quantifoil R2/1 gold EM grids or finder grids (200 mesh with holey carbon film of 2 μm hole size and 1 μm spacing) were plasma cleaned with O_2_ and H_2_ for 30 s in a glow discharge device (Gatan, Plasma Cleaner) and sterilized under UV light for 20 min in a biosafety cabinet. Subsequently, these EM grids were coated with poly-D-lysine (Gibco, A3890401) overnight, washed six times with sterilized ultrapure water, and then incu bated in HBSS for at least 6 h before seeding cells.

### Primary culture and infection of hippocampal neurons

Hippocampal neurons were isolated from P0 mice (Bi and Poo 1998) and digested with Papain (Thermo Fisher, 88285) for 30 min in a water bath at 37°C. The dissection process aimed to isolate only the hippocampus to prevent the growth of glial cells in subsequent cultures. Preferably, the dissection was performed on ice to preserve tissue activity. Following digestion, the tissues underwent two gentle washes with trituration solution (HBSS supplemented with 10% FBS, 10% BSA, 0.5 mmol/L Glutamax (Invitrogen, 35050061), and a small amount of DNase) and were mechanically triturated 15–20 times with a 1 mL tip to obtain a single-cell suspension (try to avoid bubbles). Centrifuge the suspension at 1000 r/min for 5 min at 4°C and discard the supernatant. The dissociated cells were suspended with 1 mL plating medium (DMEM/10%FBS) and seeded onto EM grids in a 35 mm dish at a low density of 70,000–100,000 cells/dish. After eight hours of seeding, the plating medium was replaced by the culture medium. The culture medium was NeuroBasal (Gibco, 10888022) supplemented with 2% B27 (Invitrogen, 17504-044) plus 0.5 mmol/L Glutamax. Subsequently, half of the culture medium was replaced with fresh culture medium every three days. Primary neurons were maintained in incubators at 37°C in 5% CO_2_ until DIV14–18, by which time the cells had fully stretched and formed a stable neuronal network.

For correlative light and electron microscopy, neurons were infected with PSD95-Dronpa at 5–7 days *in vitro* (DIV). The Dronpa cDNA was amplified from the existing plasmids in the laboratory and subcloned into pLenti-CaMKⅡ-PSD95-EGFP vector (a gift from Prof. Bi Guoqiang) to produce the pLenti-CaMKⅡ-PSD95-Dronpa plasmid. The PSD95-Dronpa lentivirus packaging was completed by Obio technology. Before virus infection, 1 mL of culture medium was removed and stored for later use. Fresh culture medium (1 mL) was then prepared and mixed with 2 μL lentivirus, which was subsequently added to the 35 mm dish and incubated for 18–24 h. Following this, the medium was replaced with a mixture of half fresh medium and half old medium stored previously. Neurons were then maintained in the incubator at 37°C with 5% CO2 until DIV14–18.

### Vitrification of neurons

Before plunge freezing, the neurons were checked for healthy and suitable for cryo-ET. BSA-coated 10 nm Gold Tracer beads (EMS, Cat. 25486) were added to the EM grids (5 μL each grid) as fiducial markers for tilt-series alignment. For cryo-CLEM, 50 nm gold nano particles (Nanopartz, AC11-50) and 0.2 μm dark red fluorescent microspheres (Invitrogen, F8807) were applied to the EM grids (5 μL each grid) as correlation markers. 0.2 μm microspheres were diluted at 1:200 and 50 nm gold particles were diluted at 1:20 in 10 nm gold tracer solution. The 50 nm gold particles were initially diluted in a 10 nm gold solution, and the particles were uniformly dispersed by ultrasonic vibration to prevent clustering. Grids were blotted from a single side (opposite to the cell side) using EMGP2 (Leica). Plunge freezing parameters were set to humidity 85%, temperature 37°C, and blot time 4 s. The grids were rapidly plunged into liquid ethane for sample vitrification and then stored in liquid nitrogen.

### Cryo-fluorescence microscopy

The cryo-fluorescence imaging system consists of a cryo-chamber, a liquid nitrogen (LN2) pump and a custom-built fluorescence microscope equipped with an EMCCD camera (iXon DV-897 BV, Andor, UK). Andor Solis Imaging software was used to control the EMCCD and acquired images. An air objective (LMPlanFLN 100×/0.8, Olympus, Japan) was used for imaging. An additional lens was inserted in front of the EMCCD to capture low-magnification images. The Cryo-chamber was cooled by continuously cold nitrogen gas and maintained at a stable cryogenic temperature of –190°C, monitored by a PT100 temperature sensor. Grids were clipped onto Autogrids (ThermoFisher, 1036171/1036173) and loaded into a custom-built sample holder capable of accommodating three Autogrid samples. A grid containing four-color fluorescent beads was used to correct the image shift between two imaging channels (supplementary Fig. S5). After loading samples to the cryo-stage, the entire grid was quickly screened to find the locations of healthy neurites with proper density using bright field imaging. The PSD95-Dronpa and the 200 nm reference beads were excited by 488 nm and 561 nm laser, respectively. An additional lens was used in the red channel to attenuate the strong red signal, enabling the capture of the green fluorescent signal. Coordinate letters were recorded under a bright field for locating the corresponding position under the electron microscope. Within two hours, 10–15 POIs were selected on one grid as soon as possible to avoid ice contamination. Subsequently, the sample was stored in a liquid nitrogen tank, and cryo-ET was performed within a week.

### Cryo-electron tomography

The vitrified cells were imaged on a Titan Krios 300 kV electron microscope (ThermoFisher Scientific) equipped with a field emission gun, a direct electron detector (Gatan, K2 camera), an energy filter and a Volta phase plate (Danev *et al.* 2014) (VPP). Tilt-series were collected from –51° to 51° at 3° increments using SerialEM software (Mastronarde 2005) in low-dose mode, with a total accumulated dose of 105 e^−^/Å^2^ , an energy filter of 20 eV, and a final pixel size of 5.432 Å. When VPP was used, the defocus was set to –1 μm; without VPP, the defocus value was maintained at –8 μm. Data collection was operated in counting and dose-fraction mode.

For correlative light and electron microscopy, middle-magnification (3600× or 4800×) montages of the square were recorded using SerialEM navigator GUI according to the previously recorded POIs of grids in the light microscope. The LM images were rotated to match the orientation of the letters on grids under the EM and the LM. Subsequently, the tilt-series were acquired at the position of PSD95-Dronpa signal.

### 3D reconstruction and segmentation

Dose-fractioned images were aligned by MotionCor2 (Zheng *et al.* 2017) to correct beam-induced motion and dose weighting. Each tilt series was down-sampled 2× with a final pixel size of 10.87 nm to enhance image contrast. Down-sampled tilt series were aligned using the default parameters in IMOD software (version 4.10.22) with the eTomo interface (Kremer *et al.* 1996; Mastronarde and Held 2017), and images alignment was performed based on gold-fiducial markers. After reconstruction in IMOD, the missing wedge was further recovered by IsoNet (Liu *et al.* 2022) to enhance the Z-direction information of the whole tomogram and to minimize the artifacts caused by the missing wedge. All the recovered tomograms with IsoNet were checked in IMOD, the one with the best contrast and the most obvious abundance to identify those synaptic gap proteins was selected to perform tomogram segmentation. We performed organelle segmentation for tomograms using the TomoSeg module in EMAN2.2 (Chen *et al.* 2017). Vesicles, gap proteins, endoplasmic reticulum (ER), mitochondria, microtubules and presynaptic or postsynaptic membranes were manually labeled with box size 64 and build training set respectively. Then each network was trained with CNN in EMAN2.2 for each organelle separately. The trained networks were applied to the whole tomogram one by one. Using UCSF Chimera (Pettersen *et al.* 2004) to filter and smooth the segmentation map, and visualize the final segmentation in 3D.

### Correlation of cryo-fLM and cryo-ET

The MultiStackReg plugin in Fiji software was used to align the two channels’ images of TetraSpeck microspheres to obtain the transformation function, which can be used to correct the image shift between PSD95-Dronpa and 0.2-μm dark red fluorescent microspheres signals. For rough correlation, 8–15 0.2-μm dark red fluorescent microspheres visible in cryo-FM andmiddle-magnification cryo-EM montage maps were manually selected at corresponding positions in MATLAB. After tilt-series reconstruction, 50 nm gold particles were used to correlate the cryo-EM map and slice image of the tomogram. Initially, the tilt series was projected in the z direction to display all the 50 nm gold particles in the tomogram in one image. Then, 6–15 50-nm gold particles at the same position in cryo-EM map and 2D projection of tomogram were chosen in MATLAB to produce a transformation function (https://github.com/ZergTBY/Bioimage/tree/master/POIregistration). Finally, the cryo-fluorescence microscopy image and tomographic slice were merged with the transformation.

## Conflict of interest

Pei Wang, Buyun Tian, Xiaojun Xu, Huiqin Luan, Yan zhang, Wenhao Sun, Liqiao Hu, Yuanyuan Li, Yuchen Yao, Weixing Li, Shuli Zhang, Xia Li, Wei Feng, Wei Ji and Yanhong Xue declare that they have no conflict of interest.
